# Controlling lightwave in Riemann space by merging geometrical optics with transformation optics

**DOI:** 10.1038/s41598-017-19015-0

**Published:** 2018-01-11

**Authors:** Yichao Liu, Fei Sun, Sailing He

**Affiliations:** 1 State Key Laboratory of Modern Optical Instrumentations, Centre for Optical and Electromagnetic Research, JORCEP, East Building #5, Zijingang Campus, Zhejiang University, Hangzhou, 310058 China; 2 Department of Electromagnetic Engineering, School of Electrical Engineering, Royal Institute of Technology (KTH), S-100 44 Stockholm, Sweden

## Abstract

In geometrical optical design, we only need to choose a suitable combination of lenses, prims, and mirrors to design an optical path. It is a simple and classic method for engineers. However, people cannot design fantastical optical devices such as invisibility cloaks, optical wormholes, etc. by geometrical optics. Transformation optics has paved the way for these complicated designs. However, controlling the propagation of light by transformation optics is not a direct design process like geometrical optics. In this study, a novel mixed method for optical design is proposed which has both the simplicity of classic geometrical optics and the flexibility of transformation optics. This mixed method overcomes the limitations of classic optical design; at the same time, it gives intuitive guidance for optical design by transformation optics. Three novel optical devices with fantastic functions have been designed using this mixed method, including asymmetrical transmissions, bidirectional focusing, and bidirectional cloaking. These optical devices cannot be implemented by classic optics alone and are also too complicated to be designed by pure transformation optics. Numerical simulations based on both the ray tracing method and full-wave simulation method are carried out to verify the performance of these three optical devices.

## Introduction

Advancement of transformation optics (TO)^1,2^ greatly enriches the connotation of optical design, and many optical devices with novel functions that were deemed impossible before have been designed and manufactured one after another, such as invisibility cloaks^3–6^, super-lenses^7–9^, concentrators^10,11^, and rotators^12,13^, etc. However, the bright future of TO is restricted by the complicated calculations and fabrication of the transformed media, especially for some devices with complex functions. Unlike classic geometrical optics (GO), which can control the light path by directly designing diverse permutations of some basic optical elements (prisms, lenses, and mirrors), TO has no particular basic optical elements. This means that we cannot directly control the light path by simply designing combinations of some basic optical elements in TO, in which the light path is indirectly modulated by the transformation media derived from coordinate transformations. The transformation media designed by TO are entirely different in various optical designs.

GO plays an important role in conventional optical design^14^, such as imaging systems^15^, illumination designs^16^, optical film designs^17^, etc. In recent years, researchers also have made some attempts to use GO for novel modern optical designs, such as ray optics invisibility cloaks^18–21^. However, due to the geometrical optics approximation, this kind of invisibility cloak only works under ray optics and performs poorly under wave optics. Many optical devices with novel functions, such as optical wormholes^22–24^, super scatterers^25–27^ and dynamic imaging^28^, cannot be designed by GO.

Taking into account the advantages and limitations of the two methods, we make the first attempt to merge classic GO design method with TO. In our mixed design method, we first use TO to introduce a parallel Riemann space in reference space, then design the light path on one Riemann space in the reference space with conventional optical elements (mirrors) by GO. By careful design of the shape and position of these conventional optical elements, novel optical devices with various fantastic functions can be obtained. Finally, we can obtain the required materials to realize the well-designed optical devices by transforming the reference space into the real space (a standard calculation in TO). Our mixed method is simple and intuitive for an optical design engineer, which can be utilized to design novel optical devices that cannot be realized by either TO or GO alone. All optical devices designed by our mixed method are impedance matched.

## Result

### Optical conformal mapping

Our mixed optical design method is based on Riemann sheets in optical conformal mapping, on which we will further design some basic elements in GO to control the light path. We first introduce some concepts on Riemann sheets and conformal mappings from complex analysis^29^. For some multi-value functions, one variable may correspond to more than one function value. To describe such one-to-many relations, Riemann sheets are introduced to describe analytical multiple-valued functions: each Riemann sheet corresponds to one value domain. Branch cuts are line segments (or curves) that connect different Riemann sheets. When conformal mapping is used in TO, it has more interesting physical meaning, and we often refer to it as optical conformal mapping^30^, which can be treated as a special case of general TO^3,31^. In TO, there are two spaces: the real space and the virtual space (often referred to as the reference space). The light and medium in the two spaces establish a corresponding relationship with the help of the coordinate transformation. Riemann sheets can be regarded as “parallel spaces” in the reference space, and the branch cut can be imagined as a bridge that connects the “parallel spaces”^32^.

In this study, to derive our mixed optical design method later. We first begin from the Zhukovski transformation in TO, which can be written as^25^:

1 $$w=z+\frac{{a}^{2}}{z}\,{\rm{or}}\,z=\frac{1}{2}(w\pm \sqrt{{w}^{2}-4{a}^{2}}).$$

**Figure 1:**
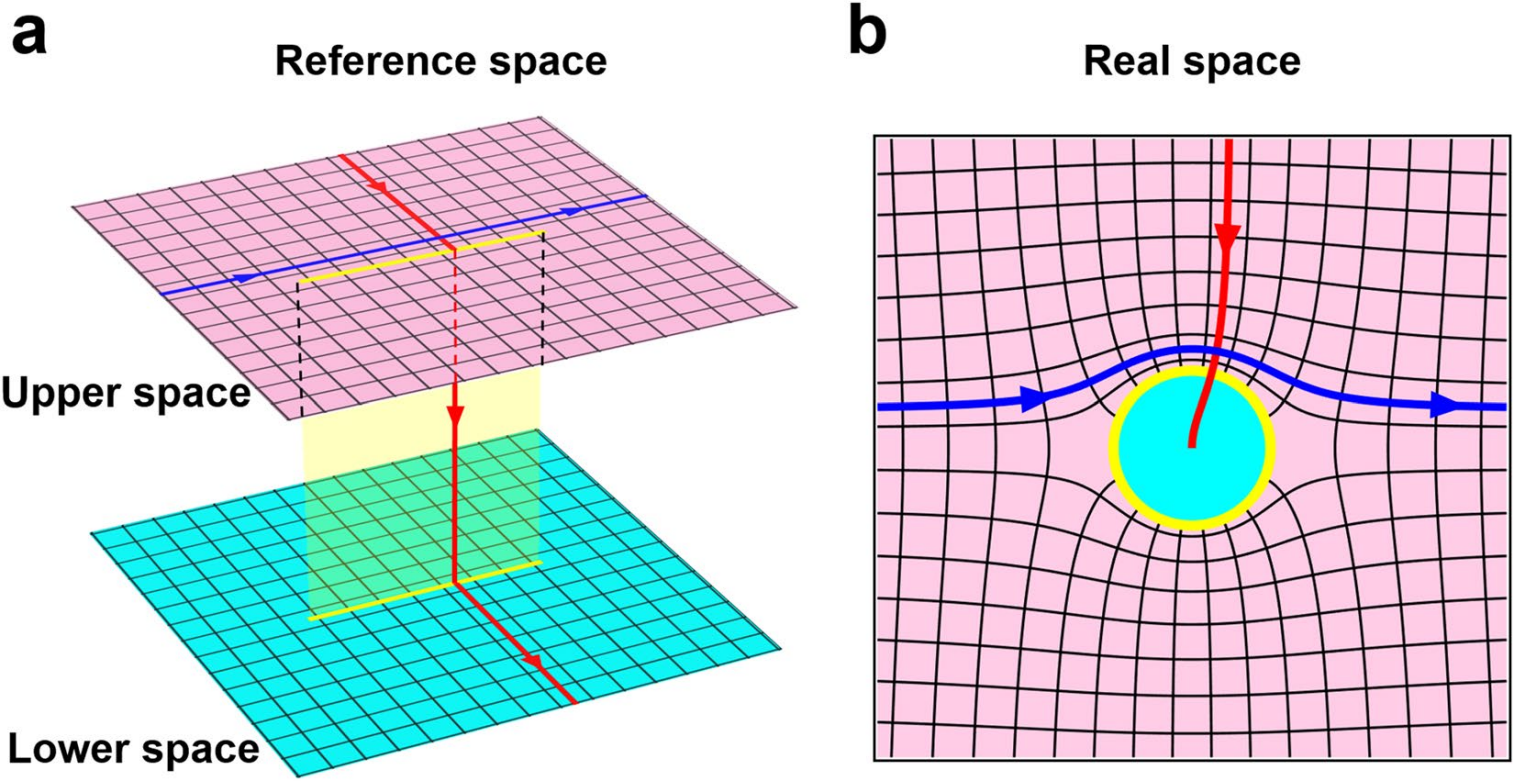
Basic function of Zhukovski transformation. (**a**) Reference space. (**b**) Real space. In (**a**), the pink plane is the first Riemann sheet (upper space), and the cyan plane is the second Riemann sheet (lower space), corresponding to the pink part (outer of the circle) and cyan part (interior of the circle) in (**b**). The red line (curve) represents a light ray which touches the branch cut, and the blue line (curve) represents another ray in the orthogonal direction, which will never touch the branch cut.

Complex coordinates *z* = *x* + *iy* and *w* = *u* + *iv* denote the coordinates in the real space and the reference space, respectively. *a* represents the size of the branch cut in the reference space (a line segment with length 4*a*) and in the real space (a circle with radius *a*). Zhukovski transformation is a kind of conformal mapping which is widely used in transformation optics and has been applied to design many novel optical devices^7,25,30^. Figure 1 shows the basic function of the Zhukovski transformation. The line segment and circle colored yellow in Fig. 1a and b are the branch cut in the reference space and the real space, respectively. The first Riemann sheet, colored pink (upper space) and the second Riemann sheet colored cyan (lower space), in the reference space in Fig. 1a correspond to the exterior and interior of the circle in the real space in Fig. 1b. Two rays propagating in orthogonal directions in the two spaces are shown as arrowed lines in Fig. 1. In the reference space, the blue ray parallel to the branch cut will never enter into the lower space and will stay in the upper space forever, while the red ray from the upper space will enter into the lower space when it touches the branch cut and will stay in the lower space forever. When we detect the trajectory of the two rays in the real space, we can see that the blue ray travels around the yellow circle and returns to the original direction; however, the red ray crosses over the circle and approaches, but never touches, the center of the circle.

To achieve the invisibility cloaking effect, some specific materials can be placed in the lower space to guide the rays back to the upper space^33^. Indeed, researchers have used well-designed lenses (such as Maxwell fish-eye lens) as a secondary design to fulfill this task and make invisibility cloaks^30,33,34^. However, the refractive index distribution by this method is very complex, and the lens in the lower space will often introduce scattering due to the impedance mismatch of the material between the two spaces. To avoid this complexity of the material, we have experimentally demonstrated a unidirectional cloaking effect by placing a mirror at the branch cut to seal up the lower space^35^. Although materials are simplified, this method also has some drawbacks. For instance, it only works under one direction. In order to overcome these difficulties, we can directly design several mirrors under the lower space, and the electromagnetic waves will be guided back to the upper space by these mirrors to achieve a bidirectional cloaking effect, which has many special features (details will be given later). Inspired by this idea, i.e. designing mirrors in the lower space by GO, we propose a mixed optical design method: Firstly, we use optical conformal mapping (equation (1)) to produce a double Riemann sheets spaces in the references space. Then, we can design mirrors by GO to guide the light on the lower Riemann sheet to achieve novel optical devices that cannot be designed by either GO or TO alone. Finally, we use TO to calculate the required materials to realize these novel devices (given in Methods). Next, we will show how to use this mixed method to design novel optical devices.

### Asymmetrical transmission

The first example is the asymmetrical transmission effect. We know that two kinds of methods are often used to achieve asymmetrical transmission. One is using nonreciprocal materials (active media) to break the time-reversal symmetry, such as nonlinear systems^7^, or applying external magnetic fields to photonic crystals^36^. The other is to break the spatial symmetry by optical systems, which often has a relatively large size, such as optical gratings^37^. Chen’s group^38^ abandoned the conventional optical system and used TO to design a device with asymmetrical transmission. However, there are some limitations of this method which makes the device difficult to put into practice, such as singularities (refractive index tends to infinity) and problems related to the large size.

**Figure 2:**
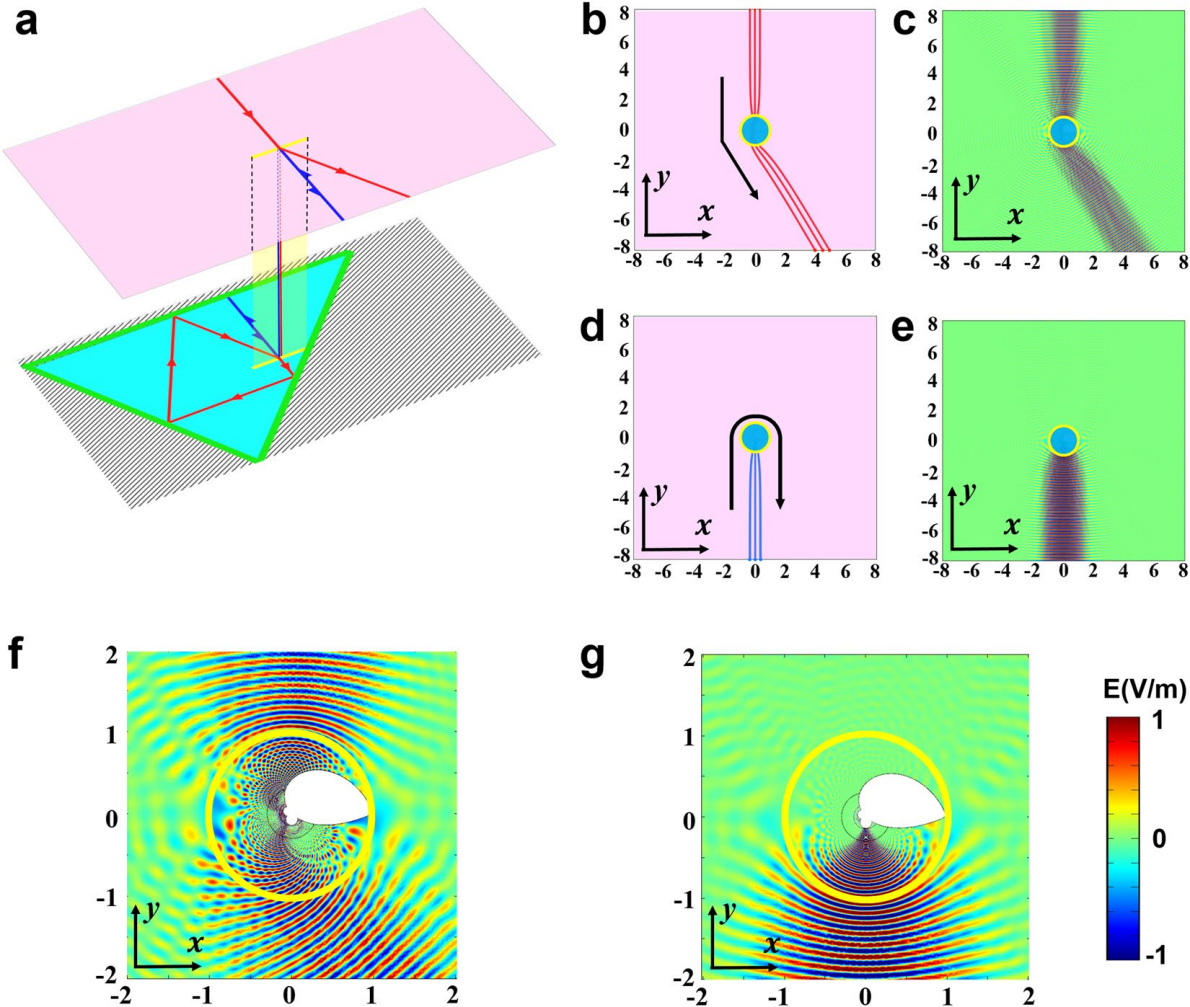
Simulation of asymmetrical transmission effect. (**a**) Light path in the reference space. The pink plane represents the upper space, and the cyan triangle represents the interior of the mirrors (green lines) of the lower space. Black hatching lines represent the region where the light will never enter. Yellow line denotes the branch cut. Red line and blue line denote two rays with opposite incident directions. (**b**,**d**) Ray tracing simulation in the real space when light is incident onto our device from top and bottom, respectively. (**c**,**e**) Corresponding electric field distribution in (**b**,**d**) by full-wave simulation, respectively. (**f**,**g**) Partial enlarged details of the black dotted box in (**c**,**e**), and the boundaries of the white region represent the mirrors. The black circles inside the branch cut (yellow circle) are just for grid meshing.

As we know, materials designed by TO are reciprocal, therefore it is hard to imagine how to use TO alone for asymmetrical transmission design. However, with well-designed mirrors in the lower space under the configuration of optical conformal mapping given in equation (1), we successfully design an optical device that can achieve the asymmetrical transmission phenomenon (see Fig. 2). Figure 2a shows how we set mirrors in the lower space in the reference space of Zhukovski transformation. The problem how to achieve an asymmetrical transmission is difficult from the perspective of TO. However, it can be converted to a quite simple problem by our mixed method: how to design mirrors by GO on the lower space in the reference space of the Zhukovski transformation to achieve asymmetrical light propagation. An optical system of three mirrors can be quickly built up in the lower space (see Fig. 2a). The blue ray propagating upward in the lower space is reflected vertically and goes back to the upper space. The red ray propagating downward in the lower space undergoes three reflections and goes back to the upper space with an angle change of *π*/6. The three mirrors form a triangle with three boundary equations (thick green lines in Fig. 2a):

2 $$\{\begin{array}{ll}v=4\sqrt{3} & u\in [-\frac{4}{\sqrt{3}}-12,4\sqrt{3}+2]\\ v=-\sqrt{3}u-4-8\sqrt{3} & u\in [-\frac{4}{\sqrt{3}}-12,\frac{-2-8\sqrt{3}}{1+\sqrt{3}}]\\ v=u-2 & u\in [\frac{-2-8\sqrt{3}}{1+\sqrt{3}},4\sqrt{3}+2]\end{array},$$

where *u* and *v* are the coordinate axes in the reference space (*w* = *u* + *iv*) as mentioned in equation (1). In the real space, the three-mirror optical system is transformed to a novel and compact device. To give a vivid description of the function of our device, a ray tracing simulation is performed, as shown in Fig. 2b and d. Black arrowed lines show the general trend of light rays. The yellow circle denotes the branch cut in the real space, and the cyan semitransparent region inside the circle denotes the lower space. In the simulation, three rays impinge on the circle from the top (red lines) and the bottom (blue lines) in Fig. 2b and d, respectively. The red rays continue their propagation only with a small deflection (*π*/6) when passing through the circle. The blue rays are totally reflected back when they enter into the circle. In order to check the function of the device under wave optics, we also give a full wave simulation (see Fig. 2c and e). Two Gaussian beams are incident from the top and bottom in Fig. 2c and e, respectively, which show good asymmetrical transmission performance of our device. The materials designed by our method in the real space are passive media, and they are isotropic and inhomogeneous. Detailed calculation of the refractive index distribution and boundaries are given in Methods.

Compared with other types of methods to achieve asymmetrical transmission^36–39^, our device has many special features, which can be summarized by: (i) Our device is a passive device, i.e. no power consumption is required, unlike other methods by non- reciprocal materials^36,39^; (ii) There is no scattering when light is incident onto our device from both directions, owing to the match of impedance. This means that there is no dissipation when light passes through our device, and therefore high efficiency can be obtained (compared with other asymmetrical transmission produced by optical gratings, where efficiency is often limited^37^); (iii) The required refractive index of our device is isotropic and has no singular point of infinity, which makes it more feasible to be put into practical application (compared with asymmetrical transmission designed by TO^38^); (iv) We can tune the angle of emergent rays by changing the position and orientation of the mirrors. Detailed discussions and simulations of the tunability of our device are shown in supplementary materials.

### Bidirectional focusing

**Figure 3:**
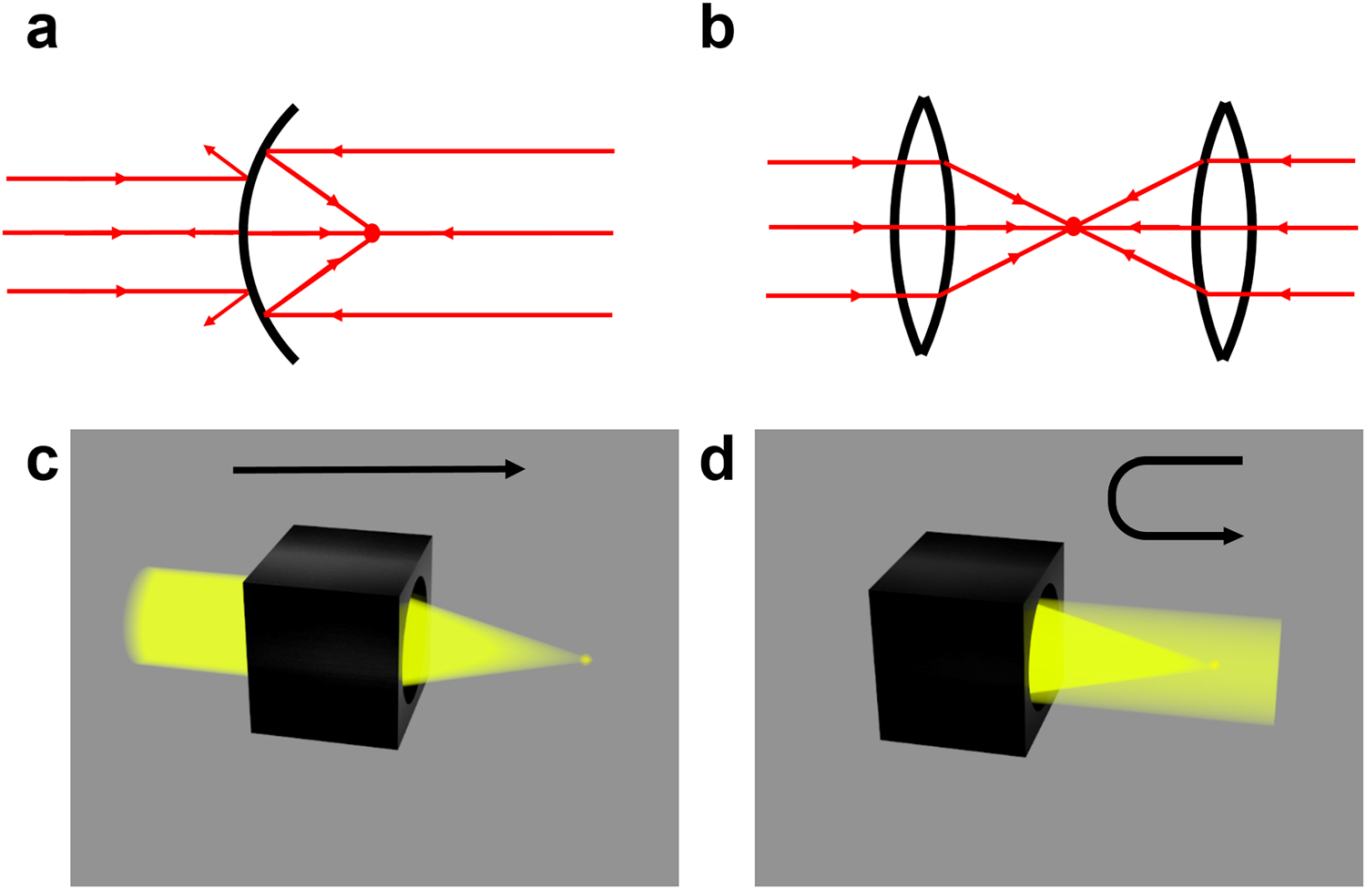
Schematic of bidirectional focusing. (**a**) Conventional parabolic mirror. (**b**) Conventional two-lens system. (**c**) Transmission-type focusing. (**d**) Reflection-type focusing. The black box is our device, which combines these two types of focusing and obtains bidirectional focusing. Note that beams from two opposite sides share the same focal point.

We continue to illustrate our mixed method by the second example: the bidirectional focusing effect. We know that a conventional lens/mirror can only focus light beams incident from one direction to its focal point. For example, as shown in Fig. 3a, the parabolic mirror can only focus parallel light from one side to its focal point. However, parallel light incident onto the parabolic lens from the opposite side will be blocked (i.e. cannot be focused). In order to focus two beams from opposite directions onto a common focal point at the same time, one possible way is to place two lenses at the opposite side of one common focal point (Fig. 3b). However, the total size of this system is very large. Can we design one compact lens to make the beam from one side focused when it passes through the lens, and the beam from the opposite side is reflected back by the lens and focused onto the same focal spot (see Fig. 3c and d)?

**Figure 4:**
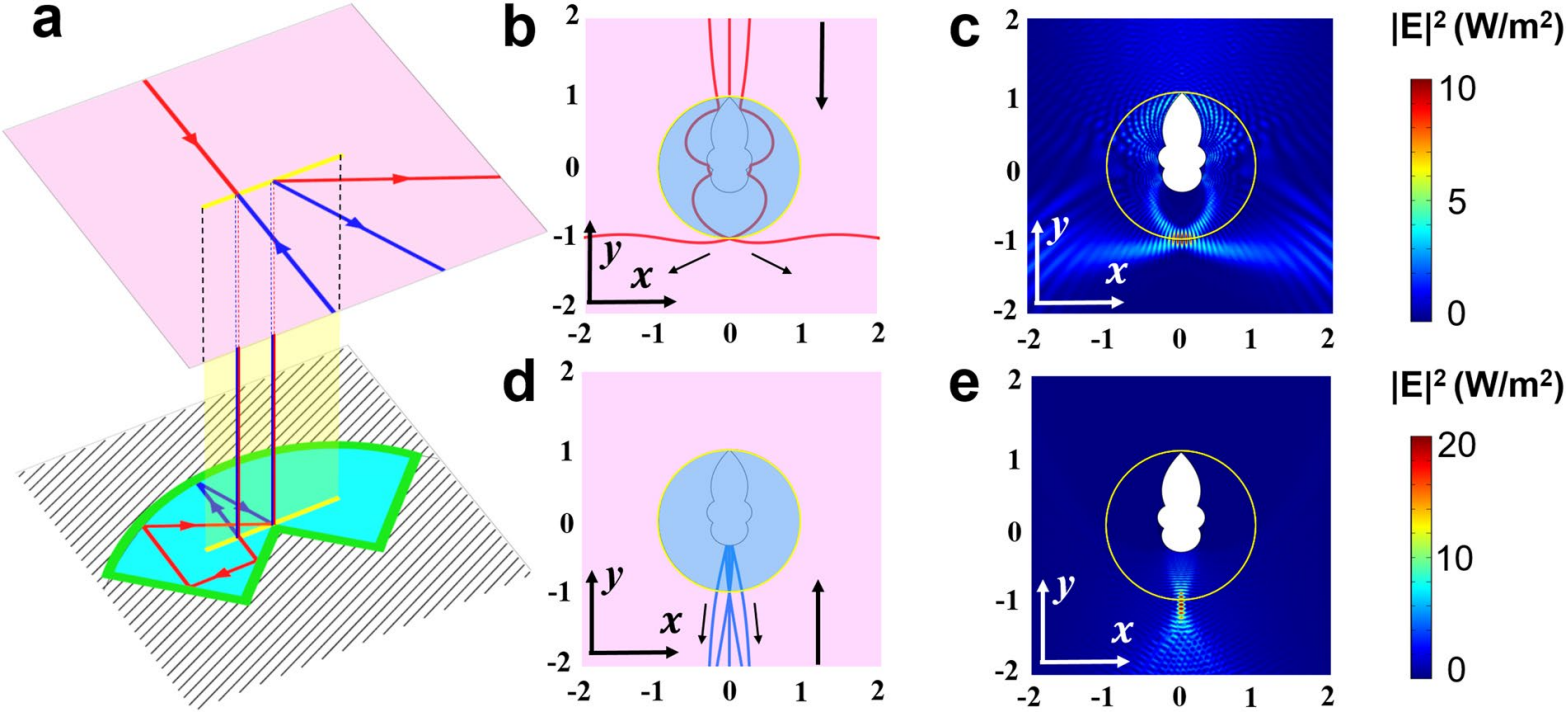
Simulation of bidirectional focusing effect. (**a**) Light path in the reference space. The pink plane represents the upper space, and the cyan triangle represents the interior of the mirrors of the lower space. Yellow line denotes the branch cut. Red line and blue line denote two rays with opposite incident directions. (**b**,**d**) Ray tracing simulation in the real space when light is incident onto our device from top and bottom, respectively. (**c**,**e**) Distribution of the square of electric field in the real space by full wave simulation when a Gaussian beam is incident onto our device from top and bottom, respectively.

Here, we use the mixed method to achieve bidirectional focusing shown in Fig. 3c and d. We also begin with two spaces (upper and lower space) in the reference space by the Zhukovski transformation (see equation (1) and Fig. 1). Then, we construct the light path by GO in the lower space. Finally, we compress the system by transforming the reference space to the real space to obtain the required refractive index distribution and mirrors’ arrangement of our device. Figure 4a shows the light path designed by GO in the reference space of the Zhukovski transformation. Besides using some plane mirrors, we also need some curved mirrors to achieve a bidirectional focusing effect. The blue ray propagating upward in the lower space is reflected back by a parabolic mirror and focuses to a point, while the red ray propagating downward in the lower space changes to the opposite direction (goes to the parabolic mirror) via two flat mirrors, then is reflected back and focuses to the same point as the blue ray. The boundary of the mirror system is determined by the following equations (colored by thick green lines in Fig. 4a):

3 $$\{\begin{array}{ll}v=u & u\in [0,2]\\ v=-u & u\in [-2,0]\\ v=u-4 & u\in [2,-5+3\sqrt{10}]\\ v=-u-4 & u\in [5-3\sqrt{10},-2]\\ v=-{u}^{2}/10+5/2 & u\in [5-3\sqrt{10},-5+3\sqrt{10}]\end{array}.$$

The above equations represent five mirrors, including one parabolic mirror and four flat mirrors. A ray tracing simulation is also used to detect the ray trajectory in the real space. In order to see clearly, the partially-enlarged drawings are given in Fig. 4b and d. Black arrowed lines denote the general trend of light rays. In the simulation, three rays impinge on the circle from the top (red lines) and the bottom (blue lines), respectively. The red rays pass through the circle and focus to a point, then diverge. The blue rays are reflected back when they enter into the circle and focus to the same point as the red rays. In order to check the function of the device under wave optics, we give a full wave simulation, and the partially enlarged drawings are plotted in Fig. 4c and e. We can see that two incident Gaussian beams from the top and bottom, respectively, focus to the same point, which validates the bidirectional focusing function of our device. The materials of this device are inhomogeneous passive media, which has the same refractive index distribution as the asymmetrical transmission device. The only difference is the boundary of mirrors (see Methods for detail).

This special device designed by our mixed method can achieve bidirectional focusing and an asymmetrical transmission effect at the same time, the advantages of which can be summarized as follows: (i) The required material of our device is isotropic, without any singular points of infinity. (ii) Our device can promote the utilization of the energy. A conventional lens or mirror only utilizes light from one direction, and a light beam from the opposite direction is reflected or focused to another point, while our device can guide the light beam from the opposite direction to focus at the same point, which means it doubles the energy utilization. (iii) The focal length of our lens can also be tuned by changing the shape and position of the mirrors. The tunability of the focal point of our device is shown in supplementary materials.

### Bidirectional cloaking

As a final example, we show how to use our mixed method to achieve a bidirectional cloaking effect. Invisibility cloaks by optical conformal mapping are theoretically studied^34,35,39^; bidirectional cloaks can be realized by placing some specified materials in the lower space. However, no experimental realizations of bidirectional full-space cloaks based on conformal mapping are reported due to the complexity of material. A unidirectional cloaking effect can be obtained by sealing up the lower space, which simplifies the material greatly and can be fabricated in the laboratory^33^. Here we will give the details on how to use our mixed method to design bidirectional full-space cloaks.

**Figure 5:**
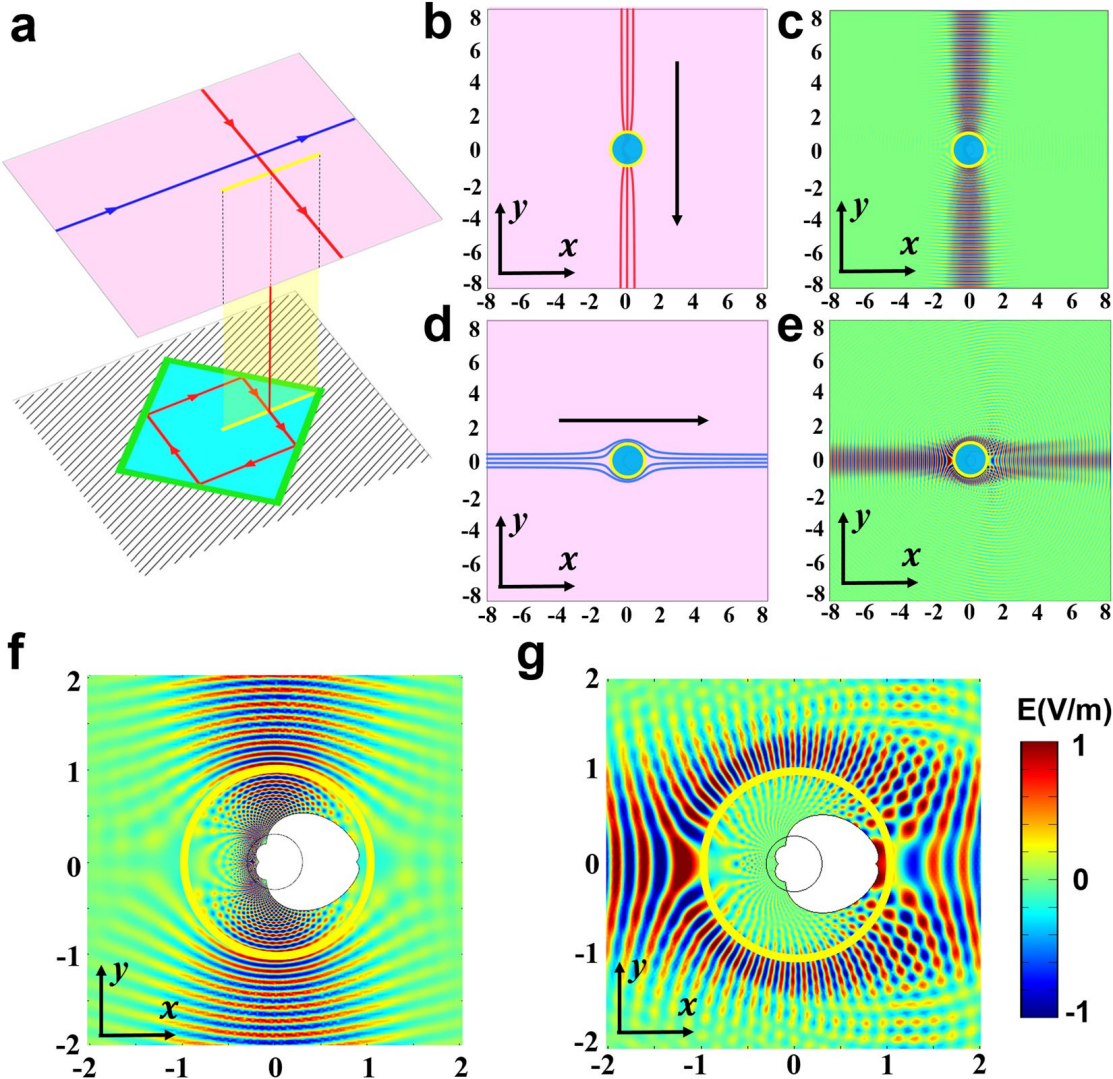
Simulation of bidirectional cloaking effect. (**a**) Light path in the reference space. The pink plane represents the upper space, and the cyan triangle represents the interior of the mirrors of the lower space. Yellow line denotes the branch cut. Red line and blue line denote two rays with orthogonal incident directions. (**b**,**d**) Ray tracing simulation in the real space. (**c**,**e**) Corresponding electric field distributions for (**b**,**d**) by full wave simulation, respectively, in the real space. (**f**,**g**) Partial enlarged details of (**c**,**e**), and the boundaries of the white region represent the mirrors.

Zhukovski transformations can achieve a unidirectional cloaking effect for light beams parallel to the branch cut (see Fig. 1a and ref.^3^]). To achieve a bidirectional full-space cloaking effect, we can simply add mirrors in the lower space of the reference space to make the device also invisible for light beams in the perpendicular direction. A four-mirror optical system is built up in the reference space of the Zhukovski transformation, as shown in Fig. 5a. Red rays perpendicular to the branch cut will pass through a loop when they enter into the lower space and then return to the upper space in the original direction. The four mirrors form a square with four boundary equations (colored by thick green lines in Fig. 5a):

4 $$\{\begin{array}{ll}v=u-2 & u\in [-2,2]\\ v=-u+2 & u\in [-2,2]\\ v=u+6 & u\in [-6,-2]\\ v=-u-6 & u\in [-6,-2]\end{array}.$$

Figure 5b and d are the ray tracing simulation results of the bidirectional cloak. Black arrowed lines denote the direction of light rays. The yellow circle denotes the branch cut in the real space, and the cyan semitransparent region inside the circle denotes the lower space. In the simulation, three rays impinge on the circle from the top (red lines) and the left (blue lines). The red rays cross over the circle, and the blue rays travel around the circle. Although rays with different directions have different ways of passing through the circle, they finally return to their original direction. We also verify the function of the bidirectional cloak under full wave simulation. Figure 5c and e represent two incident Gaussian beams from the top and left, respectively. Satisfactory cloaking effects are obtained with both of the two directions. Note that black hatching lines in Fig. 5a represent the region where the light will never enter, i.e. the free space region where concealed objects are.

We should note that the cloak is not perfect for waves due to the phase delay, which is caused by the additional path in the lower Riemann sheet and the phase change of *π* caused by the reflection on the mirror. This makes it only work at some discrete frequencies, see ref.^40^ for detail. Some scattering in Fig. 5e (not obvious) arises from a small fraction of the Gaussian beam with *y* component of wave vector, which passes though the branch cut and is reflected almost vertically to the upper space. Despite this small flaw, we give a new method for bidirectional cloaking, in which we don’t need to add complex materials in the lower space as was the case before.

**Figure 6:**
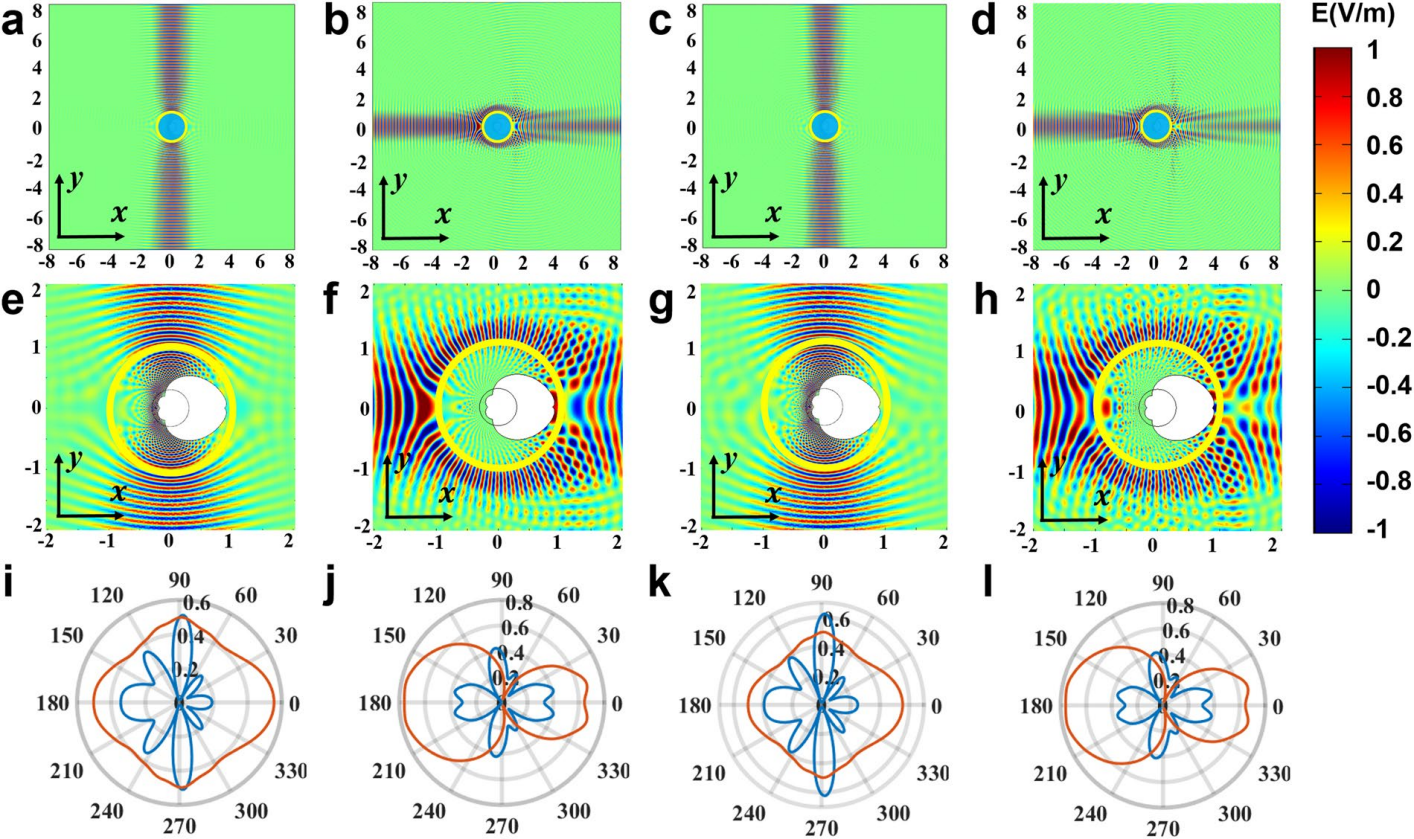
Eliminate the two zero-index points of the cloak. (**a**) and (**b**) Are for the original cloak. (**c**) and (**d**) Are for the truncated cloak by replacing any refractive index of less than 0.5 with 0.5. (**e**–**h**) Partial enlarged details of (**a**–**d**), and the boundaries of the white region represent the mirrors. (**i**–**l**) Far field patterns of the bare PEC (colored red) and the cloaks (colored blue) corresponding to (**a**–**d**) but under plane wave stimulations.

The refractive index of this device is isotropic and inhomogeneous (same as the other two devices discussed above) and has finite positive value everywhere except two zero-index points (see Method for detail). For real implementation, metamaterials from microwave to optical frequency^41–44^ could be used to realize the index. We usually truncate the cloak by replacing the refractive index of less than 0.5 with 0.5 (see ref.^7^), which makes the cloak feasible for realization in a broad operating band after a further introduction of some background material to make all the refractive index above unity. Figure 6 shows the corresponding simulation results. The truncated cloak (Fig. 6c and d) has nearly the same cloaking effect as the original one (Fig. 6a and b). In Fig. 6a–h, we use Gaussian beam in the simulation to avoid the phase delay problem since all waves enter into the lower Riemann sheet. For the far field scattering patterns in Fig. 6i–l, we use plane waves as in some previous studies^45,46^. We choose an eigen frequency of *λ* = 2*a* to minimize the effect of phase mismatch caused by the phase delay. From Fig. 6k and l we find the truncated cloak does not reduced the cloaking performance obviously compared with the original cloak (Fig. 6i and j).

Or bidirectional full-space cloak is impedance matched (i.e. no scattering is produced when light touches the branch cut) because there are both free spaces in upper and lower spaces, where we only set mirrors (PEC boundaries). The impedance matching condition is unachievable for previous methods, where inhomogeneous materials are placed in the lower space^7^. The impedance match we mentioned here means the impedance match between the upper and lower Riemann sheets at the branch cut (the circle with radius *a* in the physical space), not at the truncation boundary. Although the directions of the two beams in our design are orthogonal, we can make our invisibility cloak work under arbitrary incident angles with some modifications of the mirrors. We can even have an invisibility cloaking effect for three beams with different incident angles, and detailed discussions and simulations are shown in supplementary materials.

## Discussion

TO is a powerful optical design method which gives indirect control of light path by using complex materials that reflect the curved geometry from coordinate transformations. How to find a proper coordinate transformation has attracted great attention in the research of TO, which is also a difficult step for some reverse designs by TO. However, little attention is paid to creating some designs in the reference space. As a result, the reference space is usually an empty space. Although lenses with spherical symmetry are used in some cloak designs^34,35^, the exploration of optical designs in the reference space is not rich enough because it may make the materials more complex. In this study, we propose a method which enriches the TO by adding some ray-optics designs in the reference space without producing more complex materials. Simple and clear design is one feature of our method. The only optical element of our design in the reference space is a mirror: a PEC boundary which becomes another PEC boundary with a different shape after a coordinate transformation in the real space. No complex materials are produced by our design, and the only difference is the shape of the PEC boundary. Powerful and effective design is the second feature of our method. With different shapes of the PEC boundary, light paths are totally changed, and several devices with novel functions are developed by this method.

We use the Zhukovski transformation in this method because the lower space in the reference space corresponds to the interior of the circle in the real space. Optical systems designed in the lower space are transformed into a small region (interior of the circle) in the real space; in other words, the size of optical systems is reduced substantially by the transformation. We should note that Zhukovski transformations or other optical conformal mapping is not essential in our method: any coordinate transformation can be used in the method, provided it has the characteristics we need, such as reducing size. Our method can be regarded as a transformation of a whole optical system, and it provides the optical system with many advantages in shapes and materials.

Optical conformal mapping, which is based on the form-invariance of Helmholtz equation, is valid for both polarizations in geometrical optics or only TE polarization in wave optics^47,48^. For wave optics, the transformation media designed by optical conformal mapping are only valid for TE waves, and may introduce some unexpected scatterings for TM waves. Our full-wave numerical simulations show that some devices designed by OCM still keep satisfactory performances for TM waves, especially for the cases when the sizes of these devices are much larger than the wavelength. However, if the sizes of these devices are comparable or less than the wavelength, more unexpected scatterings may appear for TM waves.

In this study, we show three optical devices with novel functions designed by our method. The common advantages of these three devices are: (1) Tunable, such as the emergent angle of asymmetric transmission, focal point of the bidirectional focusing, and the beam angle of bidirectional cloaking. All these can be tuned by changing the shape or position of the mirrors. (2) Impedance matched. We only modify the boundaries of the reference space, and no more materials are added, so the impedance match condition is satisfied under the transformation. (3) Compact structure. As mentioned above, all of the mirrors are transformed into the interior part of the circle in the real space, which leads to a compact structure of our devices. (4) Isotropic materials.

The key idea of this study is to merge geometrical optics with optical conformal mapping to establish a new theoretical method for optical design, which can make optical design much more convenient and flexible. Our attention in this study is not focused on how to improve the performance of invisibility cloaking designed by transformation optics. Directional cloaking is just one example to demonstrate how our method works. All previous invisibility cloakings designed by TO can work only for one specific direction or omni-direction. We can achieve a bi-directional cloaking or cloaking for several pre-designed directions by our mixed method, which has never been studied before.

Some previous studies on TO also introduced the concepts of GO^49,50^. However, their ideas and purposes (i.e. eikonal approximation) are totally different from those of our work. Both works mentioned above didn’t merge GO with TO to establish a new design method, they used them separately. They first designed an optical cloaking by TO, and then simplified its materials by GO. In our work, we merge GO with TO for the first time: we design TO-based devices by a GO way (i.e. arrangements of prisms, lenses, and mirrors), instead of simplifying TO-based devices by GO.

## Conclusion

We have developed a method for optical design combining TO and GO. This method inherits the powerful control of light from TO, enriches the optical design in the reference space of TO, and creates an optical system with a more compact size than conventional optical systems. Three examples of optical devices with novel functions are shown to illustrate the idea and the validity of the method, and simulation results show that our devices perform well. With the development of modern science and technology, especially research progress in metamaterials, our method will pave the way to advance modern optical system design.

## Methods

### Calculation of the parameters and boundaries of the media

The materials designed by optical conformal transformation are isotropic, and the refractive index profile can be calculated as follows:

5 $$n(z)=n(w)|\frac{dw}{dz}|.$$

Complex coordinates *z* = *x* + *iy* and *w* = *u* + *iv* denote the coordinates in the real space and the reference space, respectively. Substituting the Zhukovski transformation of equation (1) into equation (5), we get the refractive index distribution of our device:

6 $$n(z)=|1-\frac{{a}^{2}}{{z}^{2}}|.$$

**Figure 7:**
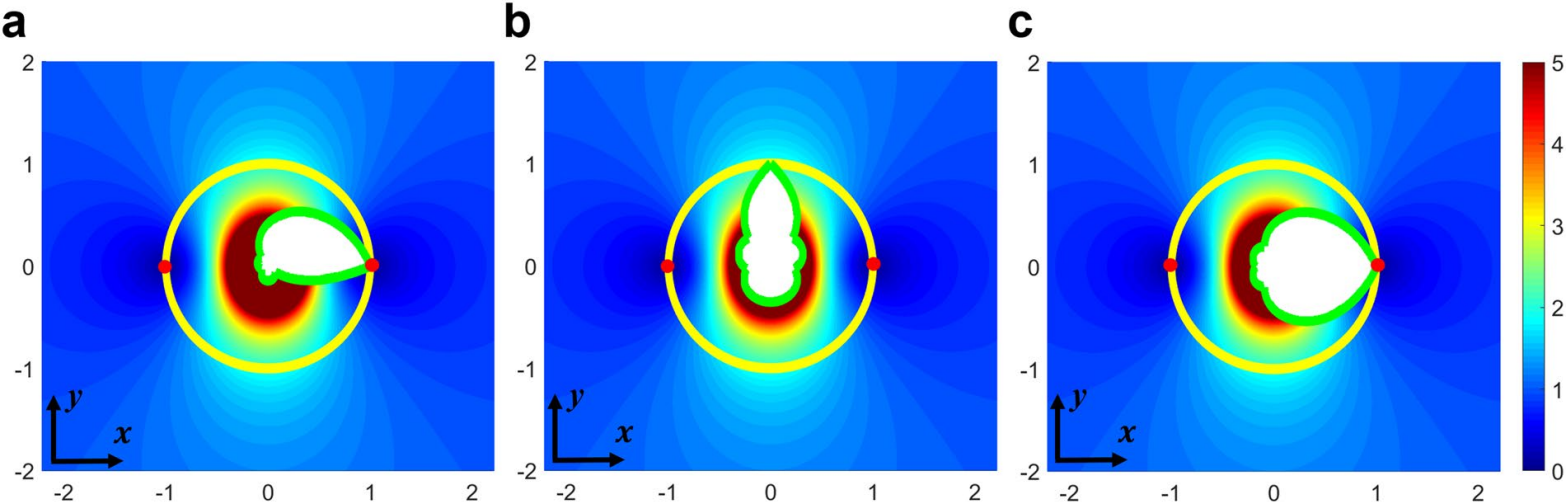
Refractive index distribution of the three devices for (**a**) asymmetrical transmission; (**b**) Bidirectional focusing; (**c**) Bidirectional cloaking. The yellow circle is the branch cut, and the green curves are the mirrors. Red dots indicate the locations where the refractive index is zero. White region is the region where light beams never touch and the material can be of arbitrary refractive index (here we fill this region with air).

This is a standard index profile used by many researches; therefore the novel functions of our device are determined by the media boundaries. Figure 7 shows the refractive index distribution and mirrors (green curves) of the three devices. In order to find the boundaries after the transformation, we use a parametric form to represent the mirrors (boundaries) in the reference space: *u* = *u*(*s*), *v* = *v*(*s*). In complex coordinates of the reference space, the mirrors can be represented as *w*(*s*) = *u*(*s*) + *iv*(*s*). Transformed into the real space, they have the form: $z(s)=\frac{1}{2}(w(s)\pm \sqrt{{w}^{2}(s)-4{a}^{2}}).$ The sign is chosen to ensure all boundaries fall into the interior of the branch cut. Finally, we get the boundaries in the real space:

7 $$\{\begin{array}{c}x(s)=\mathrm{Re}[z(s)]\\ y(s)={\rm{Im}}[z(s)]\end{array}.$$

In this study, we use flat mirrors and parabolic mirrors for the light path design. Here, we show the forms of these two types of boundaries after the transformation. Suppose these two types of boundaries have the following form before the transformation:

8 $$\{\begin{array}{c}v={c}_{1}u+{d}_{1}\\ v={c}_{2}{u}^{2}+{d}_{2}\end{array}.$$

We let parameter *s* equal *u*, and the boundaries in the real space can be deduced by equation (7); the boundary of a flat mirror is transformed to

9 $$\{\begin{array}{c}x=\frac{1}{2}\mathrm{Re}[u\pm \sqrt{-4{a}^{2}+{(u+i(u{c}_{1}+{d}_{1}))}^{2}}]\\ y=\frac{1}{2}(u{c}_{1}+{d}_{1})+\frac{1}{2}\text{Im}[u\pm \sqrt{-4{a}^{2}+{(u+i(u{c}_{1}+{d}_{1}))}^{2}}]\end{array},$$

and the boundary of a parabolic mirror is transformed to

10 $$\{\begin{array}{c}x=\frac{1}{2}\mathrm{Re}[u\pm \sqrt{-4{a}^{2}+{(u+i({u}^{2}{c}_{2}+{d}_{2}))}^{2}}]\\ y=\frac{1}{2}({u}^{2}{c}_{2}+{d}_{2})+\frac{1}{2}\text{Im}[u\pm \sqrt{-4{a}^{2}+{(u+i({u}^{2}{c}_{2}+{d}_{2}))}^{2}}]\end{array}.$$

### Simulation of the devices

In the simulation, parameters are chosen as follows: the radius of the branch cut *a* = 1 m, Gaussian beam with wavelength *λ* = 0.2 m, waist radius *w*_0_ = 5*λ* = 1 m, and waist position is in the center of the branch cut. The region with distance larger than 4*a* from the origin is truncated as air (with unity refractive index). The truncated radius 4*a* is used for general devices and wide beam width. Actually, if the width of the input beams/rays is not too large, the size of the whole device can be reduced to fit the size of the input beams/rays. For specific devices, the beam width is limited, e.g., the input beam width for a focus lens is restricted within 4*a* (otherwise some parts of the beams will not touch the branch cut and be focused). With this restriction of beam width, the focus lens can be reduced to a circle with radius 2*a*, which also have a good performance (verified by numerical simulations; not shown here). Simulations are performed by finite element analysis software Comsol Multiphysics. The ray tracing simulation is also performed using Comsol Multiphysics. We use perfect conductor boundaries as mirrors.

## Electronic supplementary material

Supplementary Information
