# Supplementary material for: Controlling lightwave in Riemann space by merging geometrical optics with transformation optics
Source: Sci Rep. 2018 Jan 11;8:514. doi: 10.1038/s41598-017-19015-0 (PMC5765110; doi:10.1038/s41598-017-19015-0)
Supplement: Supplementary file 1 — Supplementary Information [file 41598_2017_19015_MOESM1_ESM.pdf]

Controlling lightwave in Riemann space by merging geometrical optics with transformation optics

Yichao Liu[1], Fei Sun[1*] & Sailing He[1, 2, *]
1 State Key Laboratory of Modern Optical Instrumentations, Centre for Optical and Electromagnetic Research, JORCEP, East Building #5, Zijingang Campus, Zhejiang University, Hangzhou 310058, China
2 Department of Electromagnetic Engineering, School of Electrical Engineering, Royal Institute of Technology (KTH), S-100 44 Stockholm, Sweden
* Email: sunfei@zju.edu.cn, sailing@kth.se

1. Tuning the emergent angle of the asymmetric transmission device.

Figure S1 shows the optical design on the lower space in the reference space of the Zhukovski transformation. The yellow line segment with length 4*a* (*a* = 1 in the design) is the branch cut. Three mirrors (green line segments) forming a triangle have the boundary equations (l_1, l_2, l_3) as shown in Fig. S1. Red lines and blue lines represent the trajectory of two rays with opposite incident directions. In order to reduce the size of the mirror system, here we choose parameters $h = 0$, and $d = 2/\tan(\theta)$. The only variable is the emergent angle θ. Then the mirrors can be written as:

$$\begin{cases} v = d & u \in \left[\dfrac{d-b}{k},\ d+2\right] \\ v = ku + b & u \in \left[\dfrac{d-b}{k},\ \dfrac{2+b}{1-k}\right] \\ v = u - 2 & u \in \left[\dfrac{2+b}{1-k},\ d+2\right] \end{cases} \tag{S1}$$

where $d = 2/\tan(\theta)$, $k = \tan(3\pi/4-\theta/2)$, $b = 2(2/\tan(\theta)+1)\tan(\theta)\tan(3\pi/4-\theta/2)-2$. Figure S2 gives the full wave simulation of the tuning ability of the asymmetric transmission device. All simulation parameters are the same as in the main text.

2. Tuning the focal length of the bidirectional focusing device.

Figure S3 shows the optical design of a bidirectional focusing device in the reference space. The yellow line segment represents the branch cut, red and blue arrowed lines denotes two rays with opposite incident directions, and green line segments at the same position as in the main text are four flat mirrors. By adjusting the position of the parabolic mirror, we can tune the focal point (red dot) of our device. The equation for parabolic mirror can be written as:

$$\begin{gathered} v = -\frac{u^2}{2p} + \frac{p}{2} - d \\ u \in \left[p - \sqrt{p^2 - 2p(d-4-p/2)},\ -p + \sqrt{p^2 - 2p(d-4-p/2)} \right] \end{gathered} \tag{S2}$$

where p is the focal length of the parabolic mirror, d is the focal length of our device. Here we let $p = 2d + 2\sqrt{d^2+4}$ to get a minimum size of the device. By changing length d, we can tune the focal length of the device. Figure S4 shows three different focal lengths (d = 0, 1, 2) using full wave simulation.

3. Cloaking effect for two beams with arbitrary angles.

By rotating the mirror system designed in the main text by an angle θ (see Fig. S5), we can obtain a cloaking device working for two beams with arbitrary angles. The equation for mirrors can be written as:

$$\begin{cases} v = k_1 u + c_1 & u \in [\frac{d}{2\sqrt{2}}\cos(\theta) + d\sin(\theta - \frac{\pi}{4}), \frac{d}{2\sqrt{2}}\cos(\theta)] \\ v = k_2 u + c_2 & u \in [-\frac{3d}{2\sqrt{2}}\cos(\theta), \frac{d}{2\sqrt{2}}\cos(\theta) + d\sin(\theta - \frac{\pi}{4})] \\ v = k_3 u + c_3 & \mathrm{u} \in [-\frac{3d}{2\sqrt{2}}\cos(\theta), \frac{d}{2\sqrt{2}}\cos(\theta) - d\sin(\theta + \frac{\pi}{4})] \\ v = k_4 u + c_4 & \mathrm{u} \in [\frac{d}{2\sqrt{2}}\cos(\theta) - d\sin(\theta + \frac{\pi}{4}), \frac{d}{2\sqrt{2}}\cos(\theta)] \end{cases} \tag{S3}$$

where $k_1 = k_3 = -\tan\left(\theta + \frac{\pi}{4}\right)$, $k_2 = k_4 = -\tan\left(\theta - \frac{\pi}{4}\right)$, and

$$\begin{cases} c_1 = \left(\tan(\theta + \frac{\pi}{4})\cos(\theta) - \sin(\theta)\right)\frac{d}{2\sqrt{2}} \\ c_2 = \left(-\tan(\theta - \frac{\pi}{4})\cos(\theta) + \sin(\theta)\right)\frac{3d}{2\sqrt{2}} \\ c_3 = \left(-\tan(\theta + \frac{\pi}{4})\cos(\theta) + \sin(\theta)\right)\frac{3d}{2\sqrt{2}} \\ c_4 = \left(\tan(\theta - \frac{\pi}{4})\cos(\theta) - \sin(\theta)\right)\frac{d}{2\sqrt{2}} \end{cases} \tag{S4}$$

Figure S6 shows the tuning ability of cloaking for the angles between the two beams. The angle between the two beams are 60 degree (Fig. S6 (a) and (d)), 90 degree (Fig. S6 (b) and (e)) and 120 degree (Fig. S6(c) and (f)), respectively.

4. Cloaking effect for beams from three directions.

We know that Zhukovski transformation has cloaking effect for the beam direction parallel to the branch cut. Figure S7 shows the other two directions which can also have cloaking effect by a four mirrors system. Red and blue lines represent two rays with orthogonal directions. The four mirrors have the following boundary equations:

$$\begin{cases} v = -2 & u \in [-4,\ 4] \\ v = 6 & u \in [-4,\ 4] \\ u = 4 & v \in [-2,\ 6] \\ u = -4 & v \in [-2,\ 6] \end{cases} \tag{S5}$$

Figure S8 shows the simulation result by ray tracing method and full wave simulation method.

5. Property of polarization independence

In theory, conformal mapping is derived from Helmholtz equation and only valid for geometry optics[1], i.e., it is the refractive index rather than the impedance that has been taken into consideration. For waves, we must use general transformation optics theory[2] to find the accurate parameter expressions (tensors of permittivity and permeability). In our study, all of the electromagnetic parameters are derived for TE polarization and the designed devices are valid only for TE waves. However, we find the derived parameters for TE and TM polarizations have the same refractive index, but different impedances. Therefore, the device is valid for TM polarization only in geometry optics when using the parameters derived for TE polarization ($\varepsilon = n^2$, μ=1). After many simulations, we found they also have good performance for TM wave when the devices are much larger than the wavelength (usually the case). In this sense, our devices are working for both polarizations. Figure S9 shows both TE polarization (E field in z direction) and TM polarization (E filed in x-y plane) have good bidirectional focusing effects. Some different scatterings occur due to the geometry optics approximation (for TM wave) and different boundary conditions of PEC for TE and TM polarizations. We also found some similar effects in other devices (not shown here).

6. Simulation results of a size-reduced focusing lens (radius of 2*a*).

See Fig. S10.

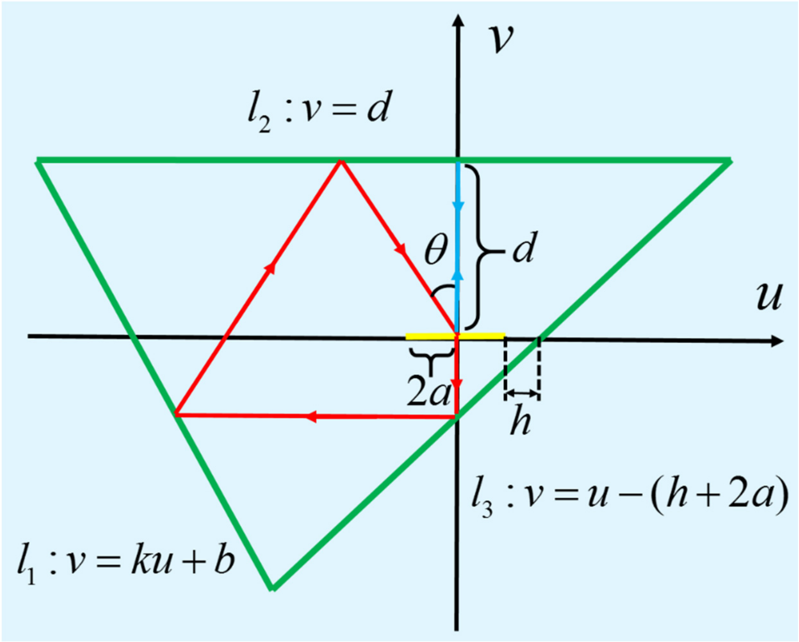

Figure S1 | Schematic of tuning ability in an asymmetric transmission device.

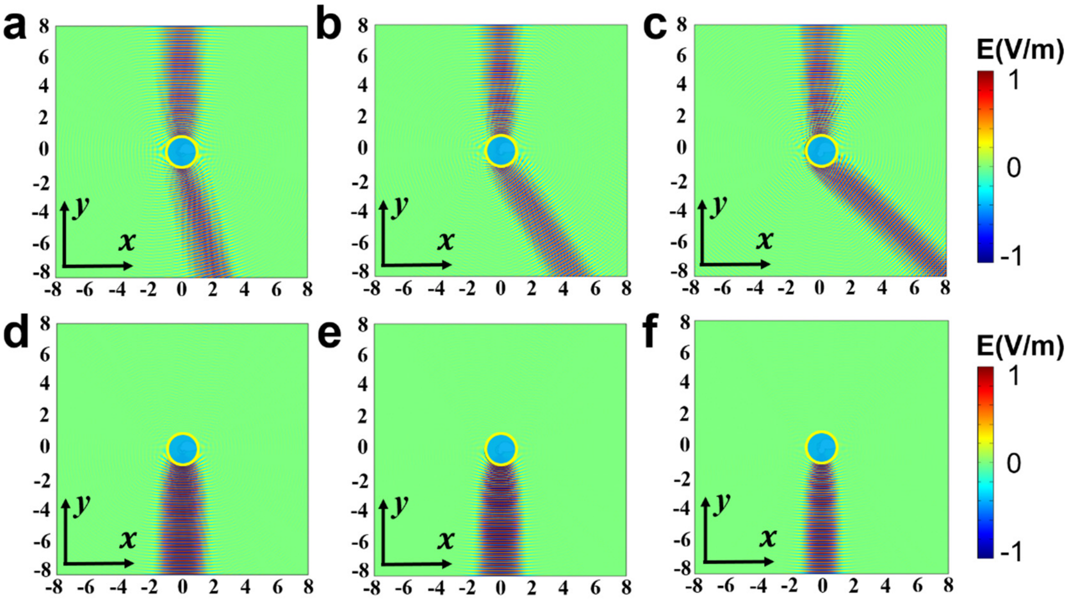

Figure S2 | Electric field distribution in the asymmetric transmission device with different emergent angle. (**a**)15 degree. (**b**) 30 degree. (**c**) 45 degree. (d)-(f) are the corresponding electric field distribution of beams incident from the bottom.

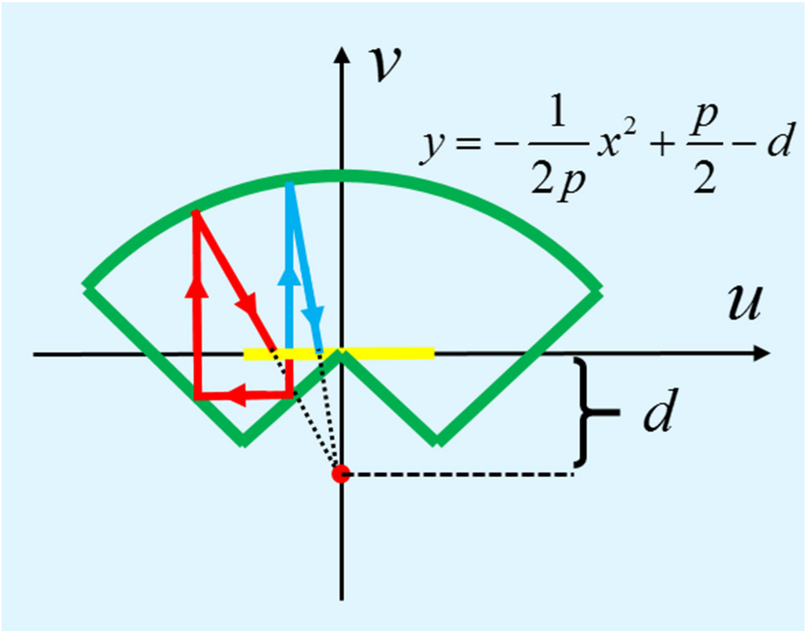

Figure S3 | Schematic of tuning ability in a bidirectional focusing device.

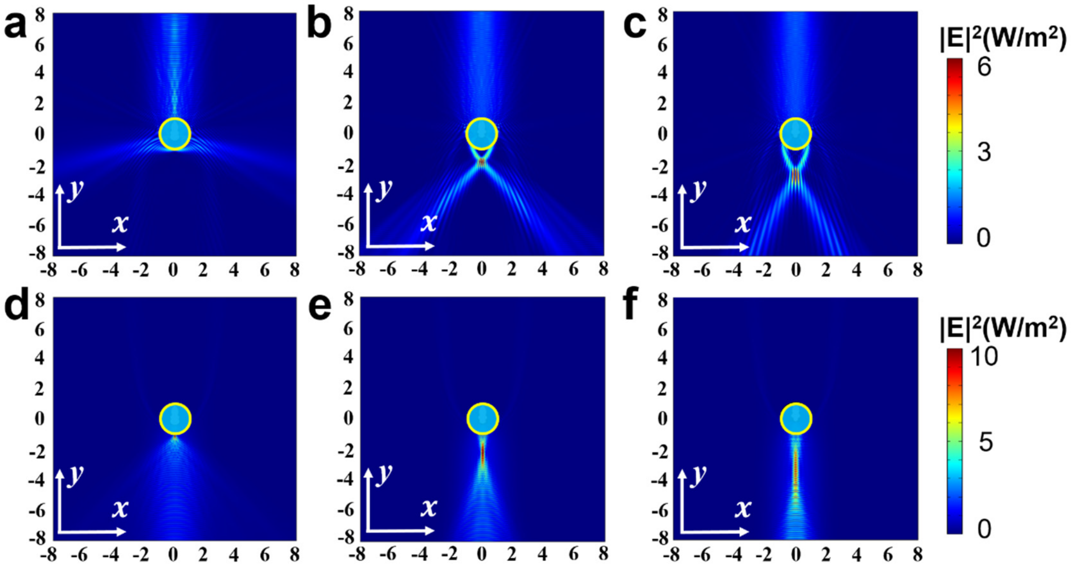

Figure S4 | Square of the electric field in bidirectional focusing device with different focal length. (**a**) and (**d**) 0 m. (**b**) and (**e**) 1 m. (**c**) and (**f**) 2 m.

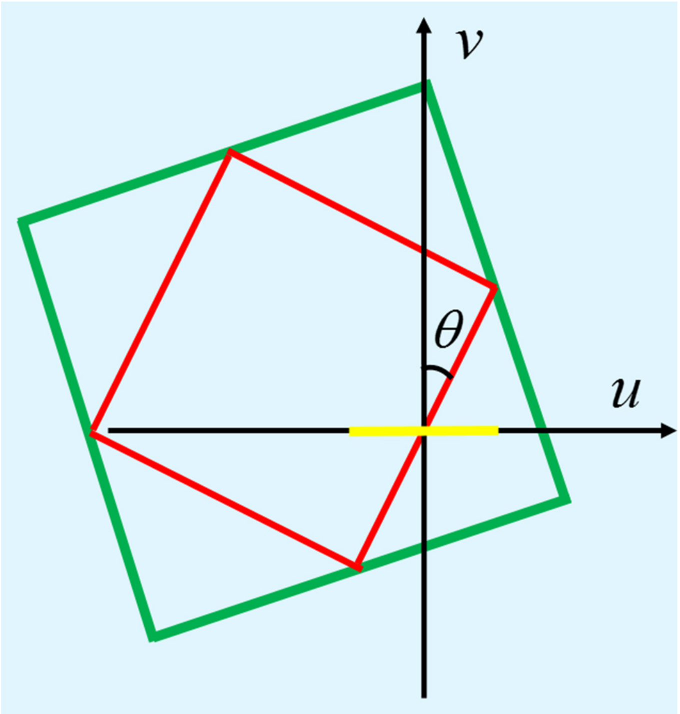

Figure S5 | Schematic of an invisibility cloak for two beams of arbitrary excitation angles.

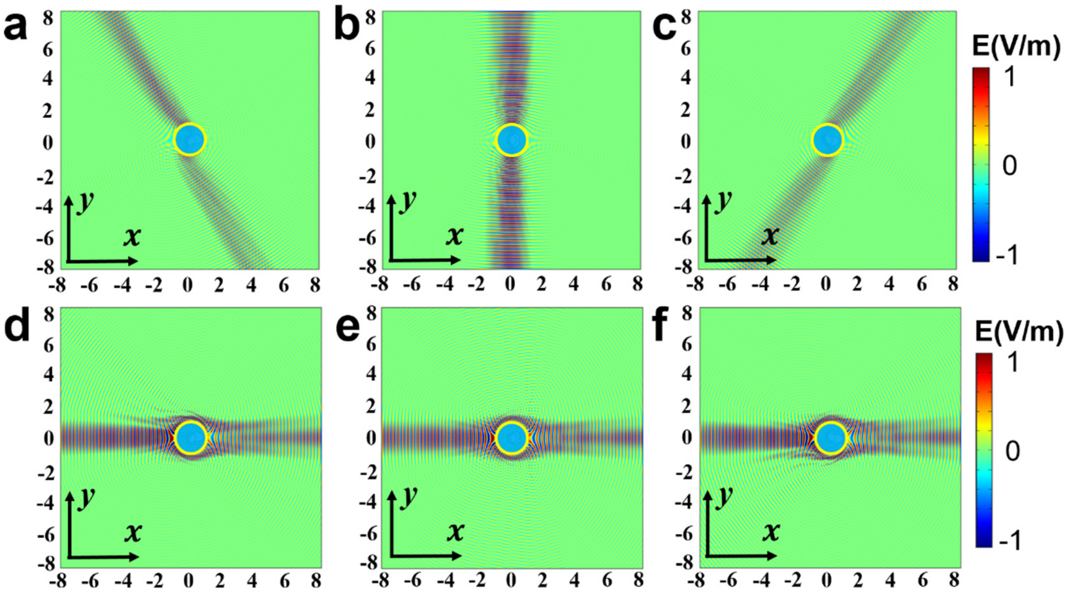

Figure S6 | Full wave simulation of our invisibility cloak for two beams with arbitrary angles. (**a**) and (**d**) with an angle of 60 degree. (**b**) and (**e**) with an angle of 90 degree. (**c**) and (**f**) with an angle of 120 degree.

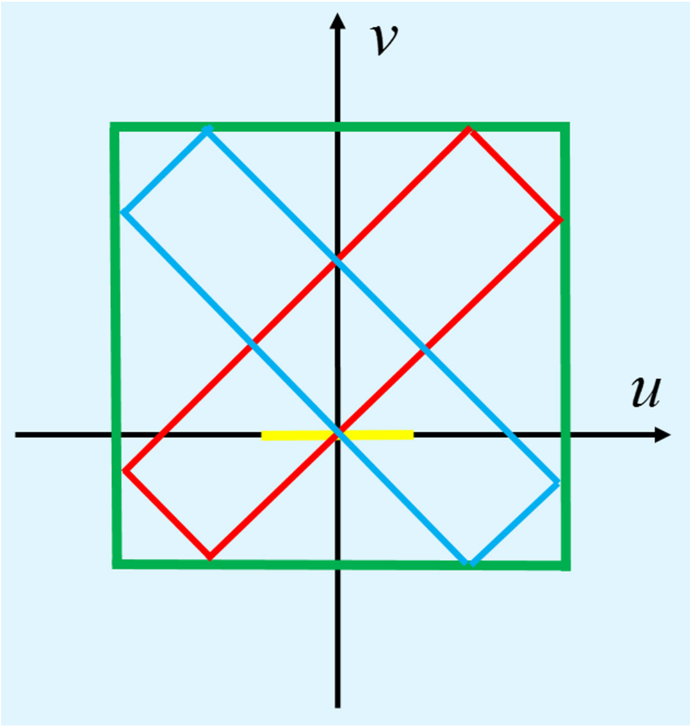

Figure S7 | Schematic of an invisibility cloak working under three incident directions.

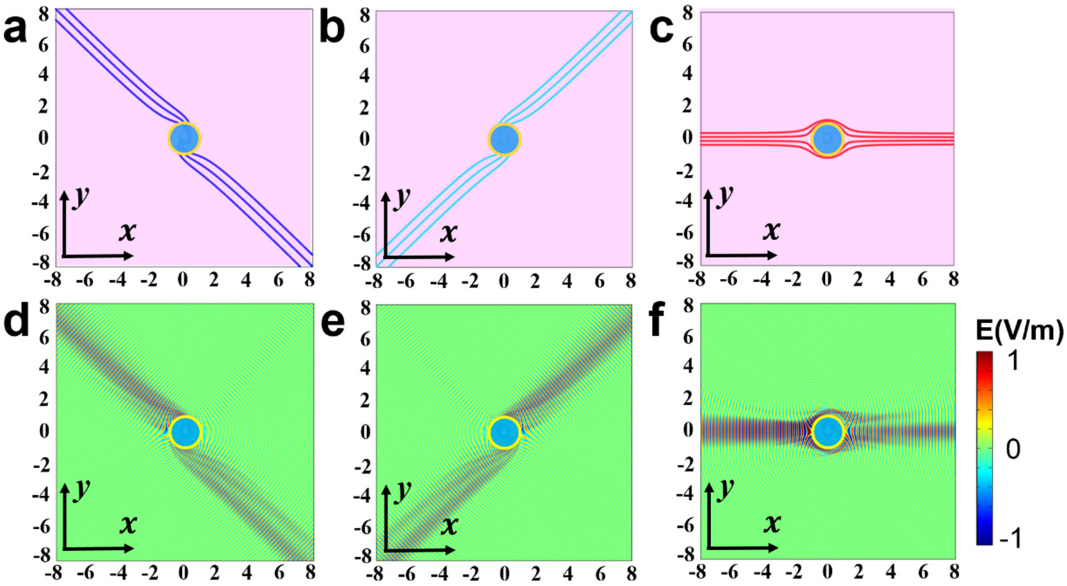

Figure S8 | Ray tracing (a-c) and full wave simulation (d-f) of the invisibility cloak device.

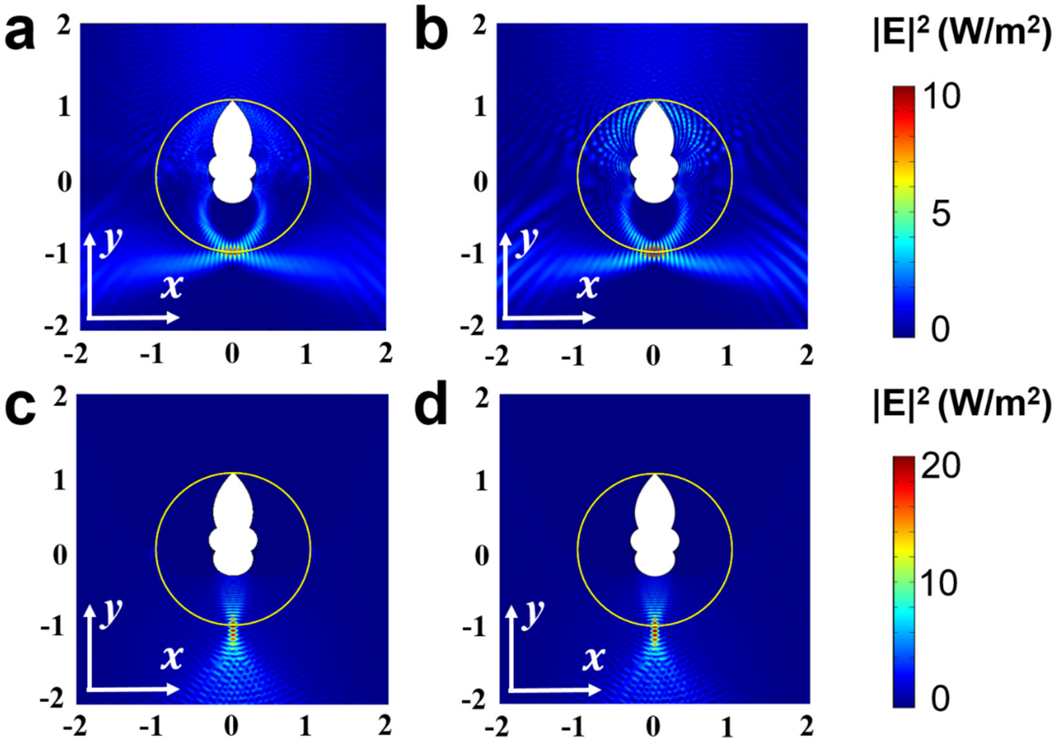

Figure S9 | Simulation of bidirectional focusing effect. (a)(c) TM polarization. (b)(d) TE polarization.

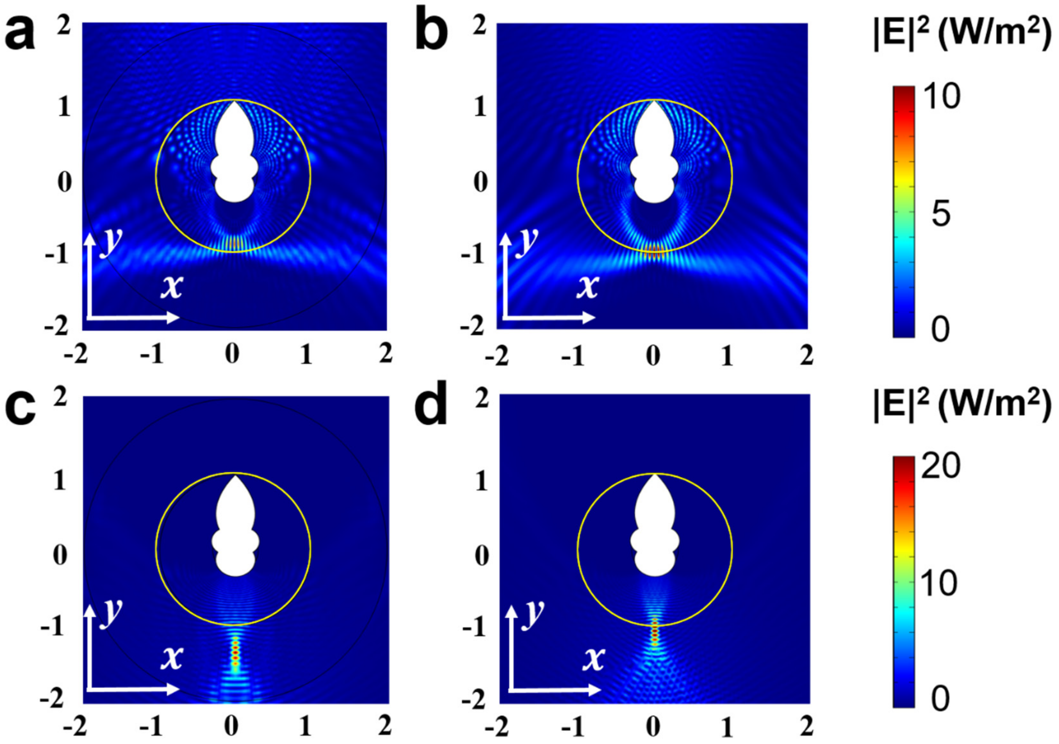

Figure S10 | Simulation of bidirectional focusing effect. (a)(c) The size of the lens is reduced to a circle with radius of 2*a***.** **(b)(d)** Original lens (a circle with radius of 4*a*)**.**

References

[1] Leonhardt, U. Optical conformal mapping. *Science* **312**, 1777-1780 (2006).

[2] Pendry, J. B., Schurig, D. & Smith, D. R. Controlling electromagnetic fields. *Science* **312**, 1780-1782 (2006).
